# CCL2: A Pro-Inflammatory Driver and Candidate Diagnostic Biomarker in Colorectal Cancer Patients

**DOI:** 10.3390/ijms27146470

**Published:** 2026-07-21

**Authors:** Carmela Nardelli, Marcella Nunziato, Federica Di Maggio, Monica Gelzo, Giuseppe Boccia, Francesco Maione, Roberto Peltrini, Fortunata Carbone, Filomena Caldora, Francesco Corcione, Giovanni Domenico De Palma, Vincenzo Pilone, Giuseppe Castaldo, Giuseppe Matarese, Francesco Salvatore, Dario Bruzzese, Lucia Sacchetti

**Affiliations:** 1 Department of Molecular Medicine and Medical Biotechnologies, University of Naples Federico II, 80131 Naples, Italy; carmela.nardelli@unina.it (C.N.); nunziato@ceinge.unina.it (M.N.); dimaggio@ceinge.unina.it (F.D.M.); gelzo@ceinge.unina.it (M.G.); giuseppe.castaldo@unina.it (G.C.); giuseppe.matarese@unina.it (G.M.); 2 CEINGE Biotecnologie Avanzate-“Franco Salvatore” S.C.A.R.L, 80145 Naples, Italy; 3 Oncological and Minimally Invasive Surgery at Clinica Mediterranea, 80122 Naples, Italy; giuseppe.boccia.29@gmail.com (G.B.); francesco.corcione@clinicamediterranea.it (F.C.); 4 Department of Clinical Medicine and Surgery, University of Naples Federico II, 80131 Naples, Italy; francesco.maione2@unina.it (F.M.); caldora@ceinge.unina.it (F.C.); giovanni.depalma@unina.it (G.D.D.P.); 5 Department of Public Health, University of Naples Federico II, Via Pansini 5, 80131 Naples, Italy; roberto.peltrini@gmail.com (R.P.); vincenzo.pilone@unina.it (V.P.); 6 Immunology Laboratory, Istituto degli Endotipi in Oncologia, Metabolismo e Immunologia “G. Salvatore”, Consiglio Nazionale delle Ricerche (IEOMI-CNR), 80131 Napoli, Italy; fortunata.carbone@ieos.cnr.it; 7 LIFE-Istituto di Ricerca e Cura Santa Lucia IRCCS, 00179 Roma, Italy

**Keywords:** colorectal cancer (CRC), CCL2, overweight/obesity, inflammatory cytokines

## Abstract

Chronic inflammation and immune remodeling are key features of colorectal cancer (CRC) development and progression, particularly because of the high level of microbiome presence. Among inflammatory mediators, CCL2 has been implicated in the recruitment of monocytes and tumor-associated macrophages, supporting its potential role as a marker of CRC-associated inflammatory remodeling. Therefore, we evaluated nine circulating inflammatory mediators, including CCL2, to assess their potential diagnostic value in patients with CRC. The study included 96 individuals, comprising 52 CRC patients and 44 healthy controls. Plasma cytokine levels were measured using the ProteinSimple Ella microfluidic immunoassay platform, and analyses were also stratified according to sex and BMI category (BMI < 25 vs. ≥25 kg/m^2^). Patients with CRC had significantly higher levels of CCL2 and IL-6 than healthy controls (*p*-value < 0.001), regardless of gender or overweight or obesity, confirming a chronic pro-tumor inflammatory profile. Among all markers, CCL2 showed strong exploratory diagnostic performance with an AUC of 0.918, 90.4% sensitivity, and 90.9% specificity (cut-off 436.5 pg/mL). This study highlights the central role of CCL2 as a candidate marker of systemic chronic inflammation associated with colorectal cancer.

## 1. Introduction

Inflammatory processes are increasingly recognized as pivotal molecular and cellular mechanisms that drive the disruption of physiological homeostasis and are associated with several chronic diseases, including the various typologies of cancer [1,2,3,4].

Recent studies across human populations have underscored the heterogeneity of inflammation, suggesting that personalized inflammatory patterns may help capture earlier the progressive molecular and cellular alterations that compromise health status and precede the development of overt disease [2,3].

Colorectal cancer (CRC) is one of the most common malignancies in Western countries, and its increasing incidence in younger adults highlights the need for improved risk stratification and early detection strategies, which remain essential to reduce CRC-related mortality [5,6,7]. Multiple factors contribute to CRC risk and progression, including genomic predisposition and other pathophysiological conditions such as overweight or obesity, inflammation and intestinal dysbiosis [8]. In this context, these alterations may sustain chronic low-grade systemic and intestinal inflammation, called inflammaging, contributing to cancer development, including CRC [9,10,11,12,13,14].

Experimental and clinical studies suggest that specific bacterial taxa and microbial-derived metabolites modulate host immune responses and shape a pro-tumorigenic cytokine milieu, providing a mechanistic link between dysbiosis, inflammation, and CRC [15].

In our previous work, we reported a CRC-associated shift in the intestinal mucosal microbiome characterized by increased abundance of *Fusobacterium nucleatum*, *Bacteroides fragilis*, and *Gemella haemolysans* [16]. Notably, *G. haemolysans* abundance positively correlated with circulating CCL2 levels, which were significantly increased in CRC patients independently of overweight or obesity, and sex/gender [16]. Together, these observations support the concept that integrating microbiome-related features with systemic inflammatory mediators may improve CRC risk stratification.

Among chemokines implicated in cancer-associated inflammation, chemokine (C-C motif) ligand 2 (CCL2) has emerged as a key mediator linking immune activation to tumorigenesis [17,18]. CCL2 can be produced by monocytes/macrophages and tumor cells, and its expression is regulated by inflammatory and oncogenic pathways, metabolites, and post-transcriptional mechanisms such as non-coding RNAs [19].

In detail, CCL2 promotes the recruitment of circulating monocytes into tumor tissues, where they may differentiate into tumor-associated macrophages and contribute to a pro-tumorigenic immune microenvironment [20]. Usually, CCL2 binds to its receptor and initiates signal transduction, stimulating cell migration and playing a role in the development and progression of various cancers, including CRC [21].

Indeed, in CRC, CCL2 has been associated with recruitment of myeloid populations (including tumor-associated macrophages and myeloid-derived suppressor cells), angiogenesis, and metastatic dissemination, making it a candidate biomarker as well as a potential therapeutic target [18,19]. Despite this biological rationale, the clinical utility of circulating CCL2 as a biomarker for CRC detection remains incompletely defined [19].

Several studies have investigated plasma cytokine signatures in CRC, suggesting that multi-analyte inflammatory profiles may discriminate between patients and healthy individuals and reflect disease stage and prognosis [22,23,24]; this strengthens the hypothesis that combining pro- and anti-inflammatory mediators may offer higher diagnostic value than single markers alone [12,23].

Based on these considerations and our previous studies, we aimed to further evaluate the diagnostic value of circulating CCL2 in CRC and investigate whether a broader inflammatory profile, including cytokines associated with Th1- and Th2-oriented immune responses, described to be involved in the adenoma–carcinoma sequence, could improve CRC diagnostic efficacy [25,26]. Given the pivotal role of CCL2 within the inflammatory tumor microenvironment [19], we assessed the pro- and anti-inflammatory immune responses. Therefore, we measured plasma CCL2 together with IL-1β, IL-12p70, IL-10, IL-2, IL-4, IL-6, IFN-ϒ, and TNF-α in 96 individuals (52 CRC patients and 44 healthy controls) in order to assess whether CCL2 alone, or in combination with other cytokine markers, could improve the ability to distinguish CRC patients from healthy controls. Importantly, the present study was designed to explore prevalently the diagnostic potential of circulating CCL2.

## 2. Results

### 2.1. Comparison of Circulating Cytokine Levels Between CRC Patients and Healthy Controls

Plasma levels of a panel of inflammatory cytokines were measured in CRC patients and healthy controls. CCL2 was measured using a single-plex cartridge, including three technical replicates, whereas the remaining eight cytokines were measured using multiplex cartridges, including two technical replicates.

A significant difference between groups was observed for IL-1β and IL-12p70, with lower levels detected in CRC patients compared with healthy controls (Table 1). CRC patients showed significantly higher circulating levels of both IL-10 and IL-6 compared with healthy controls. CCL2 plasma levels differed significantly between groups. Ratio of Geometric Means (RoGM) values greater than 1 indicate higher cytokine concentrations in CRC patients compared with controls, whereas values lower than 1 indicate reduced concentrations. No statistically significant differences were observed for IL-2, IFN-ϒ, IL-4, or TNF-α between the two groups.

Notably, all these differences remained statistically significant after adjustment for age, sex, and overweight or obesity using median regression.

**Figure 1:**
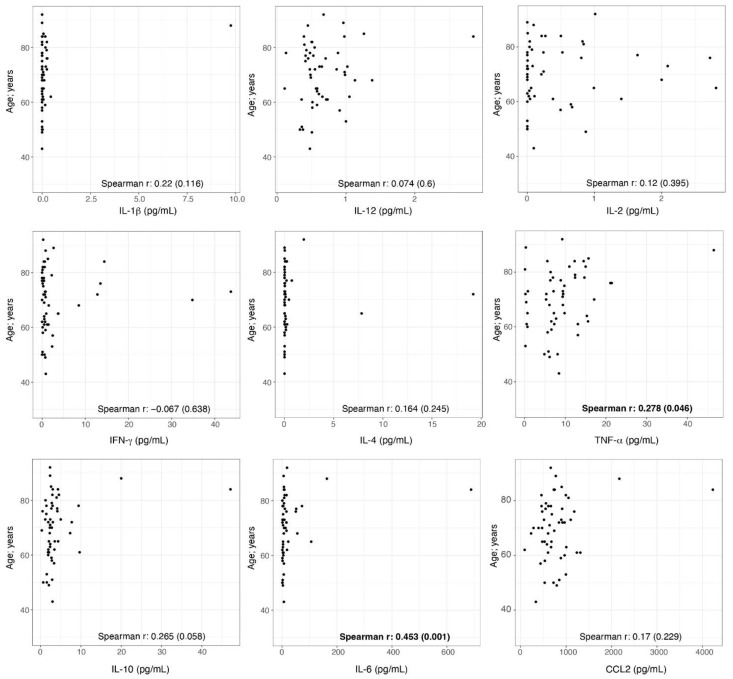
Correlation analysis between age and plasma cytokine concentrations in the CRC study population. Spearman’s rank correlation coefficients (r) and corresponding *p*-values are reported in brackets for each cytokine. Significant correlations with age were observed only for TNF-α (r = 0.278, *p*-value < 0.046) and IL-6 (r = 0.453, *p*-value < 0.001), not for CCL2. (r = 0.17, *p* value = 0.229).

To assess whether plasma cytokine levels were influenced by the age of the analyzed individuals, we evaluated the correlation between age and plasma concentrations for each cytokine in our CRC cohort. We found correlations with age only for IL-6 (r = 0.453; *p*-value = 0.001) and TNF-α (r = 0.278; *p*-value = 0.046), but not for CCL2 (r = 0.17, *p* value = 0.229), (Figure 1).

In Table 2, we report the differences observed between CRC patients and healthy controls separately for males and females, showing that the inflammatory profile associated with CRC was largely consistent across both sexes. In particular, CRC patients of both genders exhibited significantly increased plasma levels of CCL2 and IL-6 compared with healthy controls, supporting the presence of a shared tumor-associated inflammatory signature independent of sex. Similarly, IL-1β and IL-12 p70 were significantly reduced in CRC patients in both groups, suggesting a dysregulated inflammatory state associated with tumor progression.

The only apparent inconsistency between genders concerned IL-10: the trend was similar in both sexes, but statistical significance was reached only in males, likely due to the smaller sample size of the female CRC subgroup. No significant differences were detected for IL-2, IFN-ϒ, IL-4, or TNF-α in either sex. Overall, these findings indicate that the main cytokine alterations identified in CRC are maintained across genders, with CCL2 and IL-6 representing the most robust and reproducible inflammatory markers in both males and females.

Furthermore, stratified analysis by body mass index (BMI) confirmed that the inflammatory signature associated with CRC is largely independent of overweight or obesity status (Table 3). In both non-obese and overweight/obese subjects (BMI ≥ 25 kg/m^2^), CRC patients exhibited significantly higher circulating levels of CCL2, IL-6, and IL-10 compared with healthy controls, together with significantly lower IL-1β and IL-12 p70 concentrations. No significant differences were detected for IL-2, IFN-γ, IL-4, or TNF-α in either BMI. These findings suggest that the cytokine alterations observed in CRC are not merely a consequence of obesity-related low-grade inflammation, but rather reflect tumor-associated inflammatory remodeling.

We also evaluated cytokines based on the different tumor stages of the patients, but no statistically significant differences were observed, even when also pooling with or without the four G3 patients.

### 2.2. Diagnostic Performance Assessed by ROC Curve Analysis

**Figure 2:**
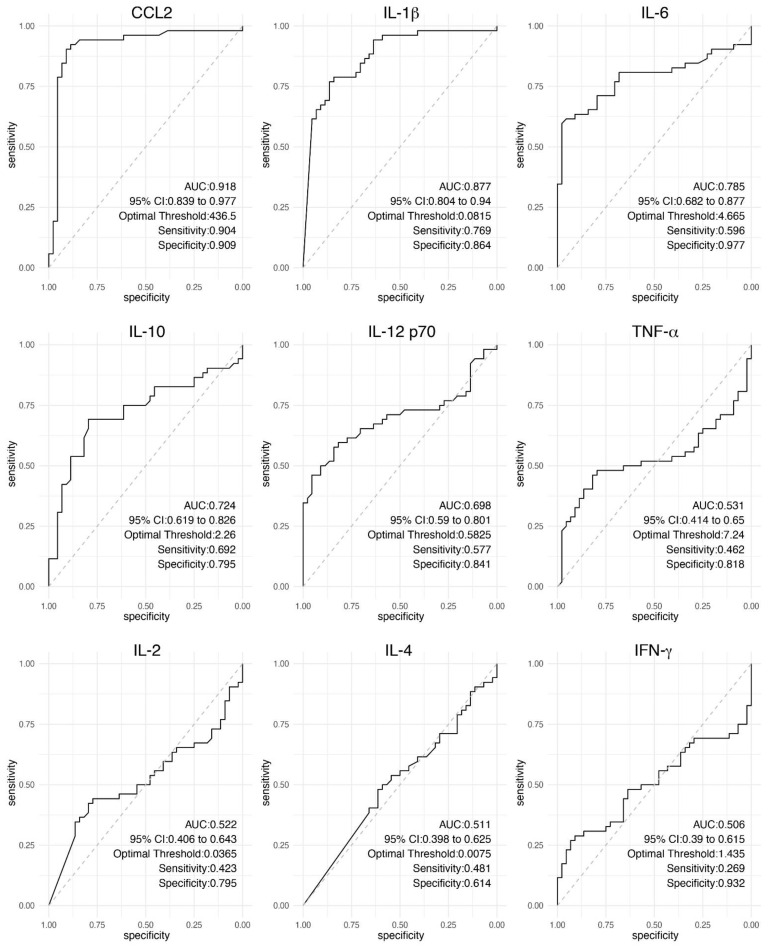
ROC curves for each measured cytokine are shown, representing them from the most significant (CCL2) to the least significant (IFN-ϒ).

To further and better evaluate the diagnostic performance of each cytokine, receiver operating characteristic (ROC) curve analysis was performed (Figure 2). This analysis allowed the assessment of sensitivity, specificity, and optimal cut-off values for each cytokine in distinguishing CRC patients from healthy controls. The results demonstrated substantial differences in diagnostic performance across the analytes.

Among the evaluated markers, CCL2 showed the highest discriminative ability, with an area under the ROC curve (AUC) of 0.918 (95% CI: 0.839–0.977). At the optimal threshold of 436.5 pg/mL, CCL2 achieved a sensitivity of 90.4% and a specificity of 90.9%, indicating high diagnostic accuracy. Similarly, IL-1β displayed strong performance with an AUC of 0.877 (95% CI: 0.804–0.94), yielding a sensitivity of 76.9% and a specificity of 86.4% at the optimal cut-off. IL-6 also demonstrated high accuracy (AUC = 0.785, 95% CI: 0.682–0.877), characterized by very high specificity (97.7%), albeit with a lower sensitivity (59.6%). IL-10 showed good discriminative capacity as well (AUC = 0.724, 95% CI: 0.619–0.826), with balanced sensitivity (69.2%) and specificity (79.5%).

Conversely, IL-12p70 exhibited only moderate diagnostic performance (AUC = 0.698), while TNF-α, IL-2, IL-4 and IFN-ϒ showed AUC values close to 0.5, indicating poor ability to distinguish CRC patients from healthy controls. Despite relatively high specificity for some of these cytokines, their low sensitivity substantially limited their utility as standalone biomarkers.

To account for potential confounding by age, sex, and BMI, the independent contribution of CCL2 and, separately, of IL-6 and IL-1β was assessed by comparing the AUC of a multivariable logistic regression model including these covariates alone with that of a model additionally including either CCL2, IL-6 or IL-1β. Although all cytokines were independently associated with CRC after adjustment for age, sex, and BMI, only CCL2 significantly improved model discrimination (ΔAUC = 0.08, *p*-value = 0.027) with a bootstrap-based estimate of optimism equal to 0.001. The estimated regression coefficients of all these models are reported in Supplementary Table S1.

## 3. Discussion

Inflammatory processes are now recognized as the molecular and cellular mechanisms that drive the disruption of physiological homeostasis, representing a hallmark of all organ diseases [1]. This dysregulation is particularly evident in chronic degenerative diseases and across the broad spectrum of human cancer. In our opinion, the overlap between features of homeostatic dysregulation suggests that these phenomena are not biologically distinct processes but represent the same degenerative trajectory [27]. However, recent research conducted on the human population has highlighted a marked heterogeneity in the inflammation profiles among individuals, leading us to consider that only a personalized assessment metric can accurately indicate the progression of molecular alterations that underlie individual health status [28,29]. This approach is essential for understanding how the inflammatory balance can influence the onset and progression of complex diseases such as CRC [30].

To explore this dynamic further, in the present study we tested a selected panel of nine cytokines and chemokines, distinguishing between pro-inflammatory and anti-inflammatory profiles. Specifically, the analysis included critical mediators of the acute response and cell recruitment, such as IL-1β, IL-12 p70, IL-2, IFN-ϒ, TNF-α, IL-6, and the chemokine CCL2 (already proposed in one of our previous studies) [16], contrasted with regulatory and anti-inflammatory factors such as IL-10 and IL-4. The use of the Ella platform, SimplePlex technology, was crucial for obtaining results characterized by high precision and rigorous reproducibility, minimizing technical variability and allowing for accurate quantification even at the lowest concentrations—a fundamental requirement for correctly mapping the state of inflammation and its correlation with CRC.

The markers showing the most pronounced increase in CRC are IL-6 and CCL2, with highly significant *p*-values (as shown in Table 1, *p*-values < 0.001). The high RoGM values for IL-6 (2.90) and CCL2 (2.44) confirm that these two molecules are primary drivers of the inflammatory process during the progression of cancer status. The increase in CCL2 is consistent with the recruitment of tumor-associated macrophages, while elevated levels of IL-6 reflect a state of chronic systemic inflammation that promotes the survival of neoplastic cells. This observation is biologically plausible, since CCL2 acts through the CCL2/CCR2 axis to recruit circulating monocytes into tumor tissues. These cells can contribute to the accumulation of tumor-associated macrophages and other myeloid populations, thereby supporting angiogenesis, immune suppression, invasion, and metastatic dissemination. In this context, elevated circulating CCL2 may reflect CRC-associated inflammatory remodeling rather than a generic inflammatory state alone.

Moreover, the absence of a significant correlation between CCL2 and age in the CRC group suggests that the elevation of plasma CCL2 may reflect inflammatory mechanisms that are independent of age, but dependent on molecular and cellular alterations, which precede overt disease [31]. Although IL-10 is an anti-inflammatory cytokine, in the context of cancer, elevated levels in CRC patients are often interpreted as a mechanism of immune escape [32]. The tumor promotes the production of IL-10 to “shut down” the activity of cytotoxic T cells, creating an immunosuppressive microenvironment that facilitates its progression [33]. Interestingly, CRC patients showed lower circulating levels of IL-1β and IL-12p70 compared with healthy controls. These differences suggest a modification of the systemic inflammatory profile in CRC; however, their biological meaning remains somehow uncertain in the absence of dedicated functional immune analyses [32].

Both genders show similar trends, but the immunosuppressive signal of IL-10 is more pronounced in males compared to females, potentially indicating a more aggressive remodeling of the immune landscape in men (Table 2).

Interestingly, although overweight or obesity is commonly associated with chronic systemic inflammation, the overall cytokine pattern remained remarkably consistent between the two BMI groups. In particular, CCL2 and IL-6 showed strong and reproducible increases in CRC patients regardless of overweight or obesity status, supporting their potential utility as robust biomarkers of CRC-associated inflammation. Overweight or obesity may act as a systemic amplifier: it does not alter the cytokine response to CRC (which remains characterized by high CCL2 and IL-6 and low IL-1β and IL-12 levels), but it exacerbates the absolute values and the immunosuppressive component. In obese individuals, the immune system must cope with a “double insult”: chronic systemic metabolic inflammation and neoplasm-induced dysregulation [34]. However, the persistence of the same cytokine alterations in normal-weight CRC patients indicates that the inflammatory signature identified in this study is primarily driven by CRC itself rather than by adiposity alone.

**Figure 3:**
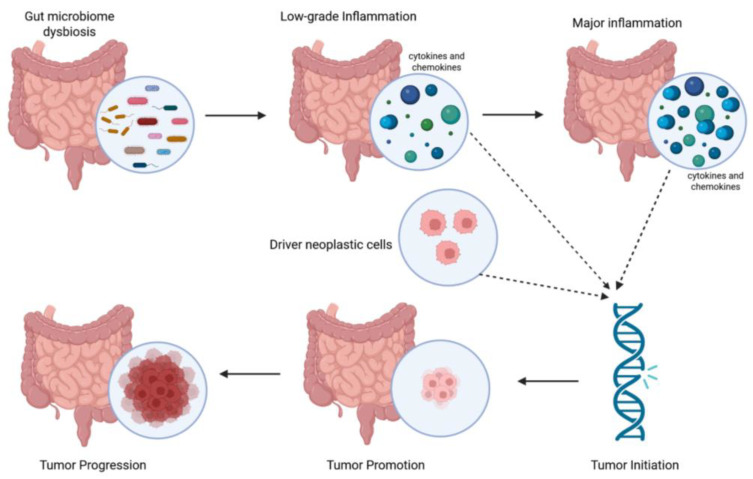
The figure shows our conceptual hypothesis that microbiome dysbiosis (e.g., *Fusobacterium nucleatum*, *Bacteroides fragilis* and *Gemella haemolisans* [16]), followed by low-grade inflammation, and then by systemic major inflammation, together with DNA damage to driver neoplastic cells, plays a pivotal role in both the initiation and progression of colorectal cancer (CRC).

This study supports our conceptual hypothesis that low-grade inflammation, rather than a major and systemic inflammation—triggered by states of dysbiosis that may be specific to different neoplastic typologies—may play a key role in the initiation and progression of colorectal cancer (Figure 3), most likely at the microenvironmental tumor sites.

From a clinical perspective, CCL2 should be considered an exploratory candidate diagnostic biomarker. The present study does not include longitudinal follow-up, survival, recurrence, or treatment–response data, and therefore cannot assess the prognostic value of CCL2.

## 4. Materials and Methods

### 4.1. Patients and Controls Enrollment

A total of 96 individuals were enrolled from the Division (UOC) of General Surgery and Minimally Invasive Oncology, Department (DAI) of Gastroenterology, Endocrinology and Endoscopic Surgery, University of Naples Federico II, at the Division (UO) of General Surgery and Minimally Invasive Oncology of Clinica Mediterranea, Naples, Italy, and at the Surgery and Medicine Department of the University of Salerno. The study population consisted of 52 patients affected by colorectal cancer (pCRC) and 44 healthy controls (HCs). Detailed characteristics of the study cohort are reported in Supplementary Tables S2 and S3.

For the pCRC group, two EDTA-anticoagulated blood tubes and one serum tube were collected the day before surgery. Among pCRC, 21 were female, and 31 were male. The mean age at diagnosis was 69.87 years (median 71.5 years). About body mass index (BMI), 33 patients had a BMI ≥ 25 kg/m^2^, while 19 patients had a BMI < 25 kg/m^2^. All pCRC underwent surgical resection of the tumor at the colorectal district. Histopathological evaluation was available 30 days after surgery. Based on tumor grading, 18 patients were classified as G1, 23 as G2, and 4 as G3; grading information was unavailable for 7 patients (see Table 4 and Supplementary Table S2).

Instead, the 44 HCs are individuals who voluntarily participated in this research study, who had no oncological or chronic degenerative diseases at the time of sampling, and who donated two tubes of EDTA blood and one tube of serum. Among HC, 24 were female, and 20 were male; the mean age was 52.31 years (median 53.0 years). The HC subjects were 28 overweight or obese (with BMI ≥ 25 kg/m^2^) and 16 normal weight. They were volunteers or recruited among those undergoing gastrointestinal controls and resulted without any cancer lesion.

For the entire study cohort, the exclusion criteria were the presence of an inflammation such as inflammatory bowel disease (IBD) or irritable bowel syndrome. The clinical and anamnestic data of each subject were collected by expert clinicians (Supplementary Table S2). 

Each patient and control enrolled signed an informed consent, according to the Helsinki Declaration 2013, and at the same time, a form was filled out containing all the clinical and family information useful for different research purposes. The study was approved by the Ethics Committee of the “Università degli Studi di Napoli Federico II”, authorization no. 233/24, December 2025 amendment; and the “Università degli Studi di Salerno”, authorization n. 50, 15 July 2015; amendment n. 141070, 26 November 2019. 

### 4.2. Sample Collection 

We collected an EDTA blood sample from HC or pCRC individuals in order to obtain a plasma sample by centrifugating for 15 min at 2500 rpm within 30 min of collection. Plasma samples were stored at −80 °C until their use. 

### 4.3. Plasma Biomarkers Measurement

The levels of plasma CCL-2/monocyte chemoattractant protein (MCP)-1, interleukin IL-1β, IL-12 p70, IL-10, IL-2, IL-4, IL-6, interferon (IFN-ϒ), and tumor necrosis factor (TNF-α) were measured by automated microfluidic immunoassay cartridges on Protein Simple Ella (Bio-Techne, Milan, Italy), according to the manufacturer’s instructions.

CCL2 was measured using a single-plex cartridge, including three technical replicates, whereas the remaining eight cytokines were measured using multiplex cartridges, including two technical replicates.

Plasma was defrosted overnight, and then 35 µL aliquots were dispensed into microcentrifuge tubes. Subsequently, the sample was centrifuged at 8000 *g* (about 10,000 rpm) for 4 min. The samples were diluted in a 1:1 ratio with the Sample Diluent. After centrifugation, 25 µL of the supernatant was carefully collected in a pre-prepared microcentrifuge tube containing 25 µL of diluent, avoiding the pellet at the bottom. Then, 50 µL of mix was loaded into the cartridge. Then, the instrument performed the entire hands-free process in about 2 h.

### 4.4. Data Analysis

Fluorescence detection and data processing on the Ella platform are fully automated and integrated within the instrument, minimizing operator-dependent variability and ensuring high reproducibility. After completion of the immunoassay, the system measures the fluorescent signal produced by the labeled detection antibody bound to the captured cytokine; fluorescence intensity is directly proportional to analyte concentration. For each run, the instrument automatically generates a calibration curve using pre-loaded standards within the cartridge, eliminating manual standard dilution steps and reducing inter-assay variability. The software automatically calculates the mean concentration and the coefficient of variation (CV%). Final cytokine concentrations are reported in picograms per milliliter (pg/mL), with automatic flagging of results below the lower limit of quantification (LLOQ) or above the upper limit of quantification (ULOQ) (Supplementary Tables S4 and S5).

### 4.5. Statistical Analysis

Statistical analyses were performed on the entire cohort, reporting continuous variables as median [25th–75th percentile] and categorical variables as counts (%). Between-group differences (CRC vs. HCs) for continuous variables (including cytokine concentrations, age, and BMI) were assessed using the Mann–Whitney U test. In addition, relative differences in cytokine concentrations between CRC patients and controls were summarized as RoGM. These were obtained by comparing mean log-transformed concentrations between groups and exponentiating the difference. Because some measurements were equal to zero, a small constant was added prior to log transformation, defined for each cytokine as half of the minimum non-zero observed value (all other analyses were instead performed on the original, untransformed cytokine values). RoGM values > 1 indicate higher concentrations in CRC patients, whereas values < 1 indicate lower concentrations. Median regression was used to assess whether between-group differences in cytokine levels persisted after adjustment for age, sex, and overweight or obesity.

The relationship between age and plasma cytokine levels was assessed for each analyte using Spearman’s rank correlation, both within the group of affected CRC patients and across the entire cohort. To evaluate the ability of each cytokine to discriminate CRC patients from controls, receiver operating characteristic (ROC) curve analysis was performed, calculating the area under the curve (AUC) with 95% confidence intervals and deriving optimal cut-off values by maximizing the Youden index, together with the associated sensitivity and specificity. Multivariable logistic regression models were fitted to assess the independent contribution of the most relevant cytokines beyond baseline clinical characteristics (age, sex, and BMI). The discriminative performance of these models was quantified by the AUC, and optimism-corrected estimates were obtained using bootstrap validation. A *p*-value < 0.05 was considered statistically significant.

## 5. Limitations

This study has some limitations that should be acknowledged. First, the case–control design and the relatively small sample size limit the generalizability of the findings. In addition, the study lacks external validation and longitudinal clinical outcome data. Controls were not age-matched with CRC patients and did not include subjects with benign colorectal diseases; therefore, the diagnostic performance of CCL2 should be considered exploratory and requires validation in larger, age-matched, multicenter cohorts, including clinically relevant benign gastrointestinal disease controls. A further limitation is the lack of direct comparison between CCL2 and established clinical markers or diagnostic tools, including CEA, CA19-9, FIT, colonoscopy-based diagnosis, and combined biomarker models. Finally, despite adjustment for the main baseline differences between cases and controls (age, sex, and BMI), residual confounding due to other clinical characteristics, including smoking habits and comorbidities, cannot be excluded.

## 6. Conclusions

In conclusion, building also on our previous evidence linking CRC-associated microbiome alterations with circulating CCL2, the present exploratory study further supports the relevance of systemic inflammatory remodeling in CRC. In our cohort, CRC patients showed a distinct circulating cytokine profile compared with healthy controls, characterized mainly by increased CCL2, IL-6, and IL-10 levels and reduced IL-1β and IL-12p70 levels. Among the analyzed cytokines, CCL2 showed the strongest exploratory discriminative performance, suggesting that it may represent a candidate marker of CRC-associated systemic inflammation.

Collectively, these findings suggest further investigation of selected circulating inflammatory cytokines, particularly CCL2, either alone or as part of a multi-marker panel, for the development of minimally invasive tools for CRC detection and patient stratification. Future studies integrating cytokine profiling, microbiome analysis, and tumor microenvironment characterization will be necessary to clarify the biological links between CRC-associated dysbiosis and systemic inflammatory responses and to assess the clinical value of CCL2 in larger prospective validating cohorts.

## Figures and Tables

**Table 1:** Cytokine plasma levels in CRC patients (n = 52) and healthy controls (n = 44).

	CRC (n = 52)	Healthy (n = 44)	*p*-Value	*BH p*-Value	RoGM
**IL-1β (pg/mL)**	**0.01 [0–0.08]**	**0.34 [0.13–0.67]**	**<0.001**	**<0.001**	**0.04**
**IL-12 p70 (pg/mL)**	**0.56 [0.46–0.88]**	**0.73 [0.64–0.9]**	**0.001**	**0.002**	**0.79**
IL-2 (pg/mL)	0.09 [0.04–0.39]	0.09 [0–0.66]	0.709	0.911	0.67
IFN-ϒ (pg/mL)	0.62 [0.18–1.51]	0.62 [0.37–0.96]	0.927	0.927	0.74
IL-4 (pg/mL)	0.01 [0–0.1]	0.02 [0–0.07]	0.856	0.927	0.96
TNF-α (pg/mL)	7.9 [5.6–12.4]	8.2 [7.5–9.6]	0.609	0.911	0.72
**IL-10 (pg/mL)**	**2.7 [2–3.9]**	**1.9 [1.5–2.2]**	**<0.001**	**<0.001**	**1.41**
**IL-6 (pg/mL)**	**6.4 [3–15.7]**	**2.3 [1.6–3.2]**	**<0.001**	**<0.001**	**2.90**
**CCL2 (pg/mL)**	**682 [528–918]**	**242 [175–313]**	**<0.001**	**<0.001**	**2.44**

All results are reported as median (25–75° percentiles). Bold characters mean significant *p*-values for the corresponding cytokines. BH: Benjamini–Hochberg false discovery rate adjustment. RoGM: Ratio of Geometric Means.

**Table 2:** Comparison of cytokine levels between healthy and CRC patients stratified by gender.

	**Males**		
	**CRC (n = 31; 60.8%)**	**Healthy (n = 20; 39.2%)**	***p*-value**
**IL-1β**	**0.01 [0–0.08]**	**0.34 [0.18–0.72]**	**<0.001**
**IL-12 p70**	**0.56 [0.45–0.7]**	**0.74 [0.6–0.89]**	**0.017**
IL-2	0.21 [0–0.87]	0.09 [0.04–0.35]	0.628
IFN-ϒ	0.63 [0.25–1.53]	0.5 [0.34–0.82]	0.556
IL-4	0.01 [0–0.11]	0.01 [0–0.04]	0.28
TNF-α	9.2 [5.9–12.4]	8 [6.7–9.5]	0.589
**IL-10**	**2.7 [2.3–3.5]**	**1.9 [1.5–2.3]**	**<0.001**
**IL-6**	**6.8 [3.1–16.2]**	**2.1 [1.4–2.9]**	**<0.001**
**CCL2**	**730 [579–913]**	**242 [172–309]**	**<0.001**
	**Females**		
	**CRC (n = 21; 46.7%)**	**Healthy (n = 24; 53.4%)**	***p*-value**
**IL-1β**	**0.01 [0–0.17]**	**0.35 [0.12–0.67]**	**<0.001**
**IL-12 p70**	**0.54 [0.44–0.9]**	**0.73 [0.64–0.91]**	**0.049**
IL-2	0.04 [0–0.33]	0.09 [0.03–0.48]	0.149
IFN-ϒ	0.57 [0.13–1.95]	0.62 [0.44–1.06]	0.707
IL-4	0.01 [0–0.11]	0.04 [0–0.14]	0.209
TNF-α	7.1 [5.1–12.8]	8.5 [7.7–9.6]	0.097
IL-10	2.8 [1.7–4.3]	1.9 [1.7–2.2]	0.106
**IL-6**	**4.9 [2.4–10.9]**	**2.6 [1.7–3.6]**	**0.009**
**CCL2**	**603 [458–966]**	**246 [188–314]**	**<0.001**

All results are reported as median (25–75° percentiles). Bold characters mean a significant *p*-value.

**Table 3:** Cytokine levels in CRC patients and controls stratified by overweight or obesity status.

	**Overweight or Obesity = NO (BMI < 25 kg/m^2^)**		
	**CRC (n = 17; 51.5%)**	**Healthy (n = 16; 48.5%)**	***p*-value**
**IL-1β**	**0 [0–0]**	**0.17 [0.08–0.49]**	**<0.001**
**IL-12 p70**	**0.51 [0.44–0.66]**	**0.69 [0.59–0.87]**	**0.012**
IL-2	0.01 [0–0.77]	0.14 [0.05–0.5]	0.285
IFN-ϒ	0.61 [0.14–1.75]	0.45 [0.36–0.63]	0.732
IL-4	0.01 [0–0.29]	0.02 [0–0.05]	0.985
TNF-α	6.1 [5.1–9]	7.4 [6–8.1]	0.397
**IL-10**	**2.4 [1.9–3]**	**1.7 [1.4–2]**	**0.024**
**IL-6**	**5.7 [1.9–14.1]**	**2.2 [1.3–3.1]**	**0.012**
**CCL2**	**666 [485–849]**	**224 [179–430]**	**0.002**
	**Overweight or Obesity = YES (BMI ≥ 25 kg/m^2^)**		
	**CRC (n = 35; 55.6%)**	**Healthy (n = 28; 44.4%)**	***p*-value**
**IL-1β**	**0.01 [0–0.16]**	**0.39 [0.25–0.77]**	**<0.001**
**IL-12 p70**	**0.58 [0.45–0.91]**	**0.76 [0.65–0.9]**	**0.017**
IL-2	0.09 [0.01–0.65]	0.09 [0.01–0.37]	0.637
IFN-ϒ	0.69 [0.25–1.53]	0.68 [0.41–1.11]	0.787
IL-4	0.01 [0–0.09]	0.02 [0–0.11]	0.756
TNF-α	9.3 [6.4–14.5]	8.9 [8.1–10.8]	0.917
**IL-10**	**2.7 [2.3–4.3]**	**2.1 [1.7–2.4]**	**0.003**
**IL-6**	**6.6 [3.5–16.2]**	**2.3 [1.7–3.8]**	**<0.001**
**CCL2**	**678 [528–966]**	**242 [166–312]**	**<0.001**

All results are reported as median (25–75° percentiles). Bold characters mean significant *p*-values.

**Table 4:** Features of the study cohort.

	CRC (n = 52; 54.2%)	Healthy (n = 44; 45.8%)	*p*-Values
Age (years)	71.5 [61.2–78] (43 to 92)	53 [44.2–61] (22 to 81)	<0.001
Sex			0.238
Male	31 (59.6)	20 (45.5)	
Female	21 (40.4)	24 (54.5)	
Body Mass Index (BMI) (Kg/m^2^)	26.9 [24–30.9] (20.7 to 36.8)	26 [24.7–34.5] (20.2 to 55.6)	0.431
Smoke			
Yes	10 (19.2%)		
No	22 (42.3%)		
Ex-Smoker	16 (30.7%)		
nr	4 (7.8%)		
Other tumors			
Yes	11 (21.1%)		
No	37 (71.1%)		
nr	4 (7.8)		
Familiarity for oncological diseases			
Yes	20 (38.5%)		
No	27 (52%)		
nr	5 (9.5)		
Histological Grade Distribution (CRC)			
G1	18 (34.6%)		
G2	23 (44.2%)		
G3	4 (7.8%)		
Missing/Not Applicable	7 (13.4%)		

nr, not reported; the age and BMI are reported as median [25°; 75° percentiles] (min and max). Histological grade distribution for CRC patients is indicated as: G1 (well-differentiated), low-grade/slow-growing; G2 (moderately differentiated), intermediate; and G3 (poorly differentiated), high-grade/fast-growing.

## Data Availability

All relevant data supporting the findings of this study are included within the article. Additional data may be available from the corresponding author upon reasonable request.

## Supplementary Materials

The following supporting information can be downloaded at: https://www.mdpi.com/article/10.3390/ijms27146470/s1.

## Abbreviations

The following abbreviations are used in this manuscript:

AUC	Area under the curve
BMI	Body mass index
CRC	Colorectal cancer
CV	Coefficient of variation
HC	Healthy control
IBD	Inflammatory bowel disease
LLOQ	Lower limit of quantification
ULOQ	Upper limit of quantification
ROC	Receiver operating characteristic
RoGM	Ratios of geometric means
