# Supplementary material for: CCL2: A Pro-Inflammatory Driver and Candidate Diagnostic Biomarker in Colorectal Cancer Patients
Source: Int J Mol Sci. 2026 Jul 21;27(14):6470. doi: 10.3390/ijms27146470 (PMC13410153; doi:10.3390/ijms27146470)
Supplement: Supplementary file 1 [file ijms-27-06470-s001.zip › ijms-4370635-supplementary.pdf]

International Journal of
Molecular Sciences

MDPI

Article

CCL2: A Pro-Inflammatory Driver and Candidate Diagnostic Biomarker in Colorectal Cancer Patients

Carmela Nardelli [1,2,†], Marcella Nunziato [1,2,†], Federica Di Maggio [1,2,†], Monica Gelzo [1,2], Giuseppe Boccia [3], Francesco Maione [4], Roberto Peltrini [5], Fortunata Carbone [6,7], Filomena Caldora [4], Francesco Corcione [3], Giovanni Domenico De Palma [4], Vincenzo Pilone [5], Giuseppe Castaldo [1,2], Giuseppe Matarese [1], Francesco Salvatore [1,2,*], Dario Bruzzese [5,*] and Lucia Sacchetti [2,*]

1 Department of Molecular Medicine and Medical Biotechnologies, University of Naples Federico II, 80131 Naples, Italy; carmela.nardelli@unina.it (C.N.); nunziato@ceinge.unina.it (M.N.); dimaggio@ceinge.unina.it (F.D.M.); gelzo@ceinge.unina.it (M.G.); giuseppe.castaldo@unina.it (G.C.); giuseppe.matarese@unina.it (G.M.); salvator@unina.it (F.S.)
2 CEINGE Biotecnologie Avanzate-"Franco Salvatore" S.C.A.R.L, 80145 Naples, Italy; sacchett@unina.it (L.S.)
3 Oncological and Minimally Invasive Surgery at Clinica Mediterranea,80122, Naples, Italy; giuseppe.boccia.29@gmail.com (G.B.); francesco.corcione@clinicamediterranea.it (F.Co.)
4 Department of Clinical Medicine and Surgery, University of Naples Federico II, 80131 Naples, Italy; francesco.maione2@unina.it (F.M.); caldora@ceinge.unina.it (F.Ca.); giovanni.depalma@unina.it (G.D.D.P.)
5 Department of Public Health, University of Naples Federico II, Via Pansini 5, 80131 Naples, Italy; roberto.peltrini@gmail.com (R.P.); vincenzo.pilone@unina.it (V.P.); dario.bruzzese@unina.it (D.B.)
6 Immunology Laboratory, Istituto degli Endotipi in Oncologia, Metabolismo e Immunologia "G. Salvatore", Consiglio Nazionale delle Ricerche (IEOMI-CNR), 80131 Napoli, Italy; fortunata.carbone@ieos.cnr.it (F.C.)
7 LIFE- Istituto di Ricerca e Cura Santa Lucia IRCCS, 00179 Roma, Italy
* Correspondence: salvator@unina.it (F.S.); dario.bruzzese@unina.it (D.B.); sacchett@unina.it (L.S.); Tel.: +39-0813737826 (F.S.)
† These authors contributed equally to this work.

Supplementary Material

Supplementary Table S1. Multivariable logistic regression models assessing the independent association of CCL2, IL-6 and IL-1β with colorectal cancer after adjustment for age, sex and BMI, and their incremental discriminative performance.

	**Adjustment Model**		**Adjustment Model + CCL2**		**Adjustment Model + IL-6**		**Adjustment Model + IL-1β**	
	**OR (95% CI)**	***p*-Value**	**OR (95% CI)**	***p*-Value**	**OR (95% CI)**	***p*-Value**	**OR (95% CI)**	***p*-Value**
Age (×5-year increase)	1.75 (1.38 to 2.22)	<0.001	1.51 (1.14 to 2.01)	0.004	1.48 (1.15 to 1.91)	0.002	1.85 (1.42 to 2.41)	<0.001
Sex; Female	0.49 (0.18 to 1.35)	0.17	0.75 (0.21 to 2.64)	0.651	0.53 (0.18 to 1.57)	0.254	0.46 (0.16 to 1.31)	0.146
BMI (kg/m^2)	1.01 (0.92 to 1.11)	0.895	1.01 (0.9 to 1.13)	0.924	0.98 (0.89 to 1.09)	0.754	1.01 (0.92 to 1.12)	0.794
CCL2 (×100 pg/mL increase)	-		1.82 (1.35 to 2.47)	<0.001	-	-	-	-
IL-6	-	-	-	-	1.35 (1.04 to 1.77)	0.027	-	-
IL-1β	-	-	-	-	-	-	0.61 (0.4 to 0.95)	0.028
Delta AUC with respect to the *adjustment model*			+0.08	0.027	+0.03	0.246	+0.02	0.245

Supplementary Table S2. Detailed characteristics of the affected studied patients (CRC).

N	ID Patient	Sex	Age	BMI	Other tumor	Smoke (Yes/NO)	Familiarity with oncological disease	Diagnosis or diagnostic hypothesis	T	N	M	Tumor grades	Number of lymph nodes
1	CRC_01	M	88	25,92	Liver metastasis	NO	YES	Adenocarcinoma of the rectosigmoid junction	T3	NX		G2	
2	CRC_02	F	61	25	NO	NO	YES	Adenocarcinoma of the right colon	T3	N2		G2	21
3	CRC_03	M	72	22	NO	YES	NO	Adenocarcinoma	T3	N1	M0	-	-
4	CRC_04	M	84	24	NO	NO	NO	Moderately differentiated adenocarcinoma	T2	N0	-	G2	28
5	CRC_05	M	77	21,8	Atrial mixoma	NO	YES	Mucinous adenocarcinoma of the colon	T4b	N0	-	G3	31
6	CRC_06	M	69	34,5	Non small cell lung cancer	EX-smoker	YES	Moderately differentiated adenocarcinoma	T3	N0	-	G2	-
7	CRC_07	F	84	30	NO	EX-smoker	YES	Moderately differentiated adenocarcinoma	T3	N0	-	G2	31
8	CRC_08	M	77	35,7	NO	EX-smoker	YES	Moderately differentiated adenocarcinoma	T1	Nx	-	G2	8
9	CRC_09	M	73	22,3	Prostate cancer	EX-smoker	NO	Moderately differentiated adenocarcinoma	T2	N0	-	G1	32
10	CRC_10	F	78	27	NO	NO	YES	Moderately differentiated adenocarcinoma	T3	N1	M1	G1	6

N	ID Patient	Sex	Age	BMI	Other tumor	Smoke (Yes/NO)	Familiarity with oncolog-ical disease	Diagnosis or diagnostic hypothesis	T	N	M	Tumor grades	Number of lymph nodes
11	CRC_11	F	57	26	NO	EX-smoker	NO	Moderately differentiated adenocarci-noma	T3	N1	-	G1	36
12	CRC_12	M	49	22,57	NO	EX-smoker	NO	Moderately differentiated adenocarci-noma	T3	N2	-	G2	28
13	CRC_13	F	61	20,7	NO	YES	NO	Moderately differentiated adenocarci-noma	T4a	N2b	Mx	G2	32
14	CRC_14	M	51	24,22	YES (nr)	YES	YES	Moderately differentiated adenocarci-noma	T3	N0	Mx	G2	40
15	CRC_15	F	73	24,03	NO	NO	NO	Moderately differentiated adenocarci-noma	T2	N0	-	G2	23
16	CRC_16	F	61	23,15	NO	NO	YES	Moderately differentiated adenocarci-noma	T3	N1a	-	G2	35
17	CRC_17	F	43	23,31	NO	NO	YES	Moderately differentiated adenocarci-noma	T3	N0	Mx	G3	36
18	CRC_18	M	68	23,94	NO	NO	NO	Well-differentiated adenocarcinoma of the colon	T1	N0	-	G1	34
19	CRC_19	M	68	31,14	NO	NO	NO	Well-differentiated adenocarcinoma of the colon	T3	N0	-	G1	26
20	CRC_20	M	73	30,11	NO	NO	NO	Poorly differentiated adenocarcinoma of the colon	T2	N0	Mx	G2	37
21	CRC_21	M	63	27,36	Liver metasta-sis	EX-smoker	YES	Well-differentiated adenocarcinoma of the colon	T4	N1	-	G1	2/21 are sites of metastasis

N	ID Patient	Sex	Age	BMI	Other tumor	Smoke (Yes/NO)	Familiarity with oncological disease	Diagnosis or diagnostic hypothesis	T	N	M	Tumor grades	Number of lymph nodes
22	CRC_22	M	81	25	NO	NO	NO	Moderately differentiated (G2) ulcerated adenocarcinoma with early infiltration of perivisceral adipose tissue	T3	N1b	Mx	G2	34
23	CRC_23	M	92	32,52	NO	EX-smoker	NO	Well-differentiated adenocarcinoma with mucinous features and extensive necrotic areas, infiltrating the muscularis propria	T2	N0	Mx	G1	24
24	CRC_24	M	58	22,3	nr	nr	nr	Well-differentiated adenocarcinoma of the colon	T3	N1b	Mx	G1	27
25	CRC_25	M	76	24,48	NO	NO	nr	Poorly differentiated adenocarcinoma of the colon	T4b	N1	Mx	G3	1/25 is site of metastasis
26	CRC_26	M	78	26,81	nr	EX-smoker	YES	Moderately differentiated colonic adenocarcinoma with full-thickness infiltration of the muscularis propria	T2	N0	Mx	G2	16
27	CRC_27	M	65	28,36	NO	EX-smoker	YES	Well-differentiated adenocarcinoma of the right colon with transmural infiltration of the bowel wall	T3	N0	Mx	G1	34
28	CRC_28	F	65	28,9	nr	nr	nr	Moderately differentiated sigmoid colon adenocarcinoma	T2	N1a	-	G2	1/15 is site of metastasis

N	ID Patient	Sex	Age	BMI	Other tumor	Smoke (Yes/NO)	Familiarity with oncolog-ical disease	Diagnosis or diagnostic hypothesis	T	N	M	Tumor grades	Number of lymph nodes
29	CRC_29	M	72	24,5	NO	nr	nr	Well-differentiated adenocarcinoma of the rectum	T1	N0	-	G1	-
30	CRC_30	F	80	25	nr	nr	nr	Conventional well-differentiated co-lonic adenocarcinoma infiltrating the bowel wall and perivisceral adipose tis-sue	T3	N1b	-	G1	2/63 are site of me-tastasis
31	CRC_31	M	76	34,95	YES (nr)	EX-smoker	NO	Moderately differentiated adenocarci-noma	T3	N0	-	G2	15
32	CRC_32	F	51	23,67	NO	YES	NO	Adenocarcinoma	T3	N0	-	G1	12
33	CRC_33	F	62	32,03	NO	EX-smoker	YES	Suspected gastric carcinoma	-	-	-	-	-
34	CRC_34	M	70	31,14	NO	EX-smoker	YES	Adenocarcinoma of the sigmoid colon associated with diverticular disease	T2	N0	-	G2	22
35	CRC_35	M	85	26,89	NO	YES	NO	Carcinoma of the transverse colon	T3	N0	-	G1	35
36	CRC_36	M	64	25,4	NO	NO	YES	Tubular adenoma exhibiting moderate dysplasia	-	-	-	-	-
37	CRC_37	M	71	31,22	NO	EX-smoker	NO	Rectosigmoid junction adenocarcinoma	T3	NX	-	G2	
38	CRC_38	M	50	36,75	NO	YES	NO	Right colon adenocarcinoma	T3	N2	-	G2	21

N	ID Patient	Sex	Age	BMI	Other tumor	Smoke (Yes/NO)	Familiarity with oncolog-ical disease	Diagnosis or diagnostic hypothesis	T	N	M	Tumor grades	Number of lymph nodes
39	CRC_39	M	59	26,2	NO	EX-smoker	YES	Rectal cancer post-chemoradiotherapy	-	-	-	G1	
40	CRC_40	F	72	31,14	NO	NO	NO	Mass at the hepatic flexure with colon biopsy performed				NA	
41	CRC_41	F	82	33,78	NO	NO	YES	Colon cancer with biopsy performed				NA	
42	CRC_42	M	79	30,07	YES (nr)	EX-smoker	NO	Neoplastic lesion, adenocarcinoma. En-doscopic biopsies of the colon				NA	
43	CRC_43	F	70	21,77	YES (nr)	NO	YES	Moderately differentiated adenocarci-noma	3	0		2	31
44	CRC_44	F	62	34,8	NO	YES	NO	Well-differentiated adenocarcinoma with infiltration of perivisceral fat	3	0	-	1	16
45	CRC_45	F	83	28	Relapse colon cancer	NO	NO	Invasive adenocarcinoma	T1	N0	-	G1	34
46	CRC_46	M	64	23,9	NO	YES	NO	Omental involvement suspected from right colon adenocarcinoma	NA				
47	CRC_47	F	88	23	NO	NO	NO	Moderately differentiated adenocarci-noma	T3	N1b	-	G2	28
48	CRC_48	F	78	33,2	NO	NO	YES	Poorly differentiated adenocarcinoma of the colon	T3	N0		G2	20
49	CRC_49	M	53	30	NO	NO	NO	Moderately differentiated adenocarci-noma	T3	N0		G2	20

N	ID Patient	Sex	Age	BMI	Other tumor	Smoke (Yes/NO)	Familiarity with oncological disease	Diagnosis or diagnostic hypothesis	T	N	M	Tumor grades	Number of lymph nodes
50	CRC_50	F	75	27	NO	NO	NO	Well-differentiated adenocarcinoma with infiltration of perivisceral fat	T3	N0	-	G1	12
51	CRC_51	M	82	28	Lung cancer	YES	NO	Poorly differentiated adenocarcinoma of the colon with invasion of the external serosal margin	T4	N1		G3	16
52	CRC_52	F	60	22,95	NO	YES	NO	Well-differentiated adenocarcinoma with invasion of the external serosal margin	T4	N0	-	G1	23

Supplementary Table S3. Information about the healthy studied patients.

N	ID	Sex	Age	BMI
1	CO_1	F	44	40.51
2	CO_2	F	31	32.00
3	CO_3	M	53	35.00
4	CO_4	F	47	37.92
5	CO_5	F	45	42.00
6	CO_6	M	45	38.50
7	CO_7	F	76	25.34
8	CO_8	F	63	26.00
9	CO_9	F	56	26.00
10	CO_10	F	61	25.00
11	CO_11	F	81	26.00
12	CO_12	M	52	26.00
13	CO_13	M	22	25.25
14	CO_14	F	72	28.70
15	CO_15	M	42	26.70
16	CO_16	F	53	25.90
17	CO_17	M	59	26.26
18	CO_18	M	55	28.00
19	CO_19	F	54	38.20
20	CO_20	F	40	30.49
21	CO_21	F	55	32.90
22	CO_22	F	58	30.60
23	CO_23	M	27	30.44
24	CO_24	M	31	55.56
25	CO_25	M	38	46.06
26	CO_26	F	40	42.87
27	CO_27	M	24	40.91
28	CO_28	M	48	39.74
29	CO_29	F	47	23.19
30	CO_30	F	52	22.50
31	CO_31	F	51	24.61
32	CO_32	M	48	24.84
33	CO_33	M	44	24.80
34	CO_34	M	66	21.31
35	CO_35	M	65	24.84
36	CO_36	F	70	24.97
37	CO_37	M	45	24.80
38	CO_38	F	57	20.20
39	CO_39	M	78	24.11
40	CO_40	F	61	23.15
41	CO_41	M	64	23.99
42	CO_42	M	66	24.00
43	CO_43	F	57	24.00
44	CO_44	F	59	24.00

Supplementary Table S4. LLOD and LLOQ of the panel used. In the table below are the typical ranges of Lower Limit of Detection (LLOD) and Lower Limit of Quantification (LLOQ) for the panels used herein, expressed in pg/mL.

Analyte	LLOD (pg/mL)	LLOQ (pg/mL)
CCL-2 (MCP-1)	~0.1–0.3	~0.3–1.0
IL-1β	~0.02–0.1	~0.1–0.3
IL-12 p70	~0.05–0.2	~0.2–0.5
IL-10	~0.02–0.1	~0.1–0.3
IL-2	~0.05–0.2	~0.2–0.5
IL-4	~0.02–0.1	~0.1–0.3
IL-6	~0.02–0.1	~0.1–0.3
IFN-γ	~0.03–0.1	~0.1–0.3
TNF-α	~0.03–0.1	~0.1–0.3

Supplementary Table S5. Number (%) of samples below the Lower Limit of Quantification (LLOQ) and equals 0 for the panels used.

	CRC		Healthy	
**Analyte**	**Below LLOQ**	**Equal 0**	**Below LLOQ**	**Equal 0**
CCL2 (MCP-1)	12 (23.1)	0 (0)	12 (27.3)	0 (0)
IL-1β	50 (96.2)	31 (59.6)	25 (56.8)	2 (4.5)
IL-12 p70	13 (25)	0 (0)	0 (0)	0 (0)
IL-10	0 (0)	0 (0)	0 (0)	0 (0)
IL-2	38 (73.1)	15 (28.8)	37 (84.1)	6 (13.6)
IL-4	48 (92.3)	20 (38.5)	41 (93.2)	15 (34.1)
IL-6	0 (0)	0 (0)	0 (0)	0 (0)
IFN-Υ	13 (25)	6 (11.5)	2 (4.5)	0 (0)
TNF-α	3 (5.8)	0 (0)	1 (2.3)	0 (0)

Disclaimer/Publisher's Note: The statements, opinions and data contained in all publications are solely those of the individual author(s) and contributor(s) and not of MDPI and/or the editor(s). MDPI and/or the editor(s) disclaim responsibility for any injury to people or property resulting from any ideas, methods, instructions or products referred to in the content.
